# Model-based evaluation of the cost effectiveness of 3 *versus* 6 months’ adjuvant chemotherapy in high-risk stage II colon cancer patients

**DOI:** 10.1177/1756284820954114

**Published:** 2020-09-16

**Authors:** Gabrielle Jongeneel, Marjolein J. E. Greuter, Felice N. van Erning, Miriam Koopman, Geraldine R. Vink, Cornelis J. A. Punt, Veerle M. H. Coupé

**Affiliations:** Department of Epidemiology and Biostatistics, Amsterdam UMC, VU University, PO Box 7057, MF F-wing, Amsterdam, 1007 MB, The Netherlands; Department of Epidemiology and Biostatistics, VU University, Amsterdam, The Netherlands; Department of Research and Development, Netherlands Comprehensive Cancer Organization (IKNL), Utrecht, The Netherlands; University Medical Center Utrecht, Utrecht University, Utrecht, The Netherlands; Department of Research and Development, Netherlands Comprehensive Cancer Organization (IKNL), Utrecht, The Netherlands; University Medical Center Utrecht, Utrecht University, Utrecht, The Netherlands; Department of Medical Oncology, University of Amsterdam, Amsterdam, The Netherlands; Department of Epidemiology and Biostatistics, VU University, Amsterdam, The Netherlands

**Keywords:** adjuvant chemotherapy, colon cancer, cost effectiveness, treatment duration

## Abstract

**Background:**

Our aim was to evaluate the cost effectiveness of 3 months’ adjuvant chemotherapy *versus* 6 months in high-risk (T4 stage + microsatellite stable) stage II colon cancer (CC) patients.

**Methods:**

Using the validated PATTERN Markov cohort model, which simulates the disease progression of stage II CC patients from diagnosis to death, we first evaluated a reference strategy in which high-risk patients were treated with chemotherapy for 6 months. In the second strategy, treatment duration was shortened to 3 months. Both strategies were evaluated for CAPOX (capecitabine plus oxaliplatin) and FOLFOX (fluorouracil, leucovorin and oxaliplatin). Based on trial data, we assumed that shortened treatment duration compared with a 6-month regimen was equally effective for CAPOX and less effective for FOLFOX. Adverse events were highest in the 6-month strategy. Analyses were conducted from a societal perspective using a lifelong time horizon. Outcomes were number of CC deaths per 1000 patients and total discounted costs and quality-adjusted life-years (QALYs) per patient (pp). Incremental net monetary benefit (iNMB) was calculated using a willingness-to-pay value of €50,000/QALY.

**Results:**

For CAPOX, the 6-month strategy resulted in 316 CC deaths per 1000 patients, 6.71 QALYs pp and total costs of €41,257 pp. The 3-month strategy resulted in an equal number of CC deaths, but higher QALYs (6.80 pp) and lower costs (€37,645 pp), leading to a iNMB of €8454 per person for 3 months *versus* 6 months. For FOLFOX, the 6-month strategy resulted in 316 CC deaths per 1000 patients, 6.71 QALYs pp and total costs of €47,135 pp. The 3-month strategy resulted in more CC deaths (393), lower QALYs (6.19 pp) and lower costs (€44,389 pp). An iNMB of −€23,189 was found for 3 months *versus* 6 months.

**Conclusion:**

Our findings indicate that 3 months’ adjuvant chemotherapy should be considered as standard of care in high-risk stage II CC patients for CAPOX, but not for FOLFOX.

## Introduction

Colon cancer is a major disease in The Netherlands. With around 10,000 new cases and 3750 deaths in 2017, it leads to a significant burden for patients, healthcare and society.^1^ To illustrate, the total healthcare costs for colon cancer patients in the Netherlands are estimated at 597 million euros in 2017.^2^ This is 0.7% of the total Dutch healthcare expenditure, and 10.2% of all expenditure on cancer care in that year. Around a quarter of the newly diagnosed colon cancer patients are classified as stage II.

The initial treatment for stage II colon cancer patients is surgical resection. Without further adjuvant treatment and depending on the tumor invasiveness, around 20% of these patients will develop a recurrence in the next 5 years.^3^ Therefore, stage II patients who are considered at high risk of developing a recurrence are eligible for adjuvant chemotherapy. In The Netherlands, only a T4 stage in combination with a microsatellite stable (MSS) tumor is considered as a selection criterion for adjuvant chemotherapy in stage II colon cancer.^4^ Using this criterion, 8.5% of the total stage II population in The Netherlands is eligible for adjuvant chemotherapy. However, clinicians may of course deviate from this recommendation. To illustrate, data of the Netherlands Cancer Registry (NCR) showed that only 21% of the stage II population with an indication for adjuvant chemotherapy actually received adjuvant chemotherapy in The Netherlands between 2015 and 2017.^5^ In addition, 4% percent of the patients without indication for adjuvant chemotherapy were treated with adjuvant chemotherapy.

Until recently, the standard adjuvant chemotherapy treatment regimen for high-risk stage II patients in The Netherlands was 6 months’ oxaliplatin with a fluoropyrimidine.^6–9^ Although this treatment lowers the risk of recurrence,^3^ approximately 60% of the treated patients suffer from peripheral neuropathy as a result of the adjuvant chemotherapy, and for some patients, the side effects are chronic.^10^ Neuropathy-related complaints can have a major impact on a patient’s quality of life.^11^ Previous studies showed that the degree of neuropathy is correlated with the duration of treatment; the percentage of patients who suffer from peripheral neuropathy halves when the treatment duration is shortened to 3 months.^10,12^ This raises the question whether the duration of the treatment can be shortened without reducing the effectiveness.

So far, two trials, both part of the International Duration Evaluation of Adjuvant Therapy (IDEA) collaboration,^10,12^ compared a 3-month to a 6-month adjuvant treatment duration in high-risk stage II colorectal cancer patients. In the SCOT trial, the authors reported a hazard ratio (HR) for recurrence of 0.99 [95% confidence interval (CI): 0.75–1.31] for a 3-month oxaliplatin-based treatment regimen compared with 6 months of treatment in high-risk stage II colorectal cancer. This suggests that a 3-month treatment duration is non-inferior to a 6-month treatment duration.^10^ In contrast, the TOSCA trial found a significant HR of 1.42 (95% CI: 1.06–1.90) for recurrence of disease when comparing 3 *versus* 6 months of treatment in stage II colon cancer. The most likely explanation for these conflicting findings is the higher number of patients treated with FOLFOX (fluorouracil, leucovorin and oxaliplatin) in the TOSCA trial (64%) compared with the SCOT trial (32%). This is supported by a pooled analysis of the stage II patients included in the IDEA trials.^13,14^ The results strongly suggest that 3 months of CAPOX (capecitabine plus oxaliplatin) is non-inferior to 6 months of CAPOX, whereas 3 months of FOLFOX is inferior to 6 months of FOLFOX.^13,14^

Based on the findings from the IDEA collaboration, opinions are shifting regarding the optimal duration of oxaliplatin-based adjuvant chemotherapy. Recently presented data showed that approximately 27% of the European clinicians prescribe 3 months of chemotherapy to high-risk stage II and III patients.^15^ Furthermore, in The Netherlands the recommended treatment duration for high-risk stage II patients is recently revised from 6 to 3 months of oxaliplatin-based chemotherapy.^4^ To carefully balance the benefits and potential harms of shortening the treatment duration, we evaluated the cost effectiveness of a 3-month adjuvant chemotherapy regimen compared with a 6-month regimen for CAPOX and FOLFOX separately in high-risk stage II colon cancer patients using the ‘Personalized Adjuvant TreaTment in EaRly stage coloN cancer’ (PATTERN) model.^16^

## Methods

### PATTERN model

**Figure 1.**
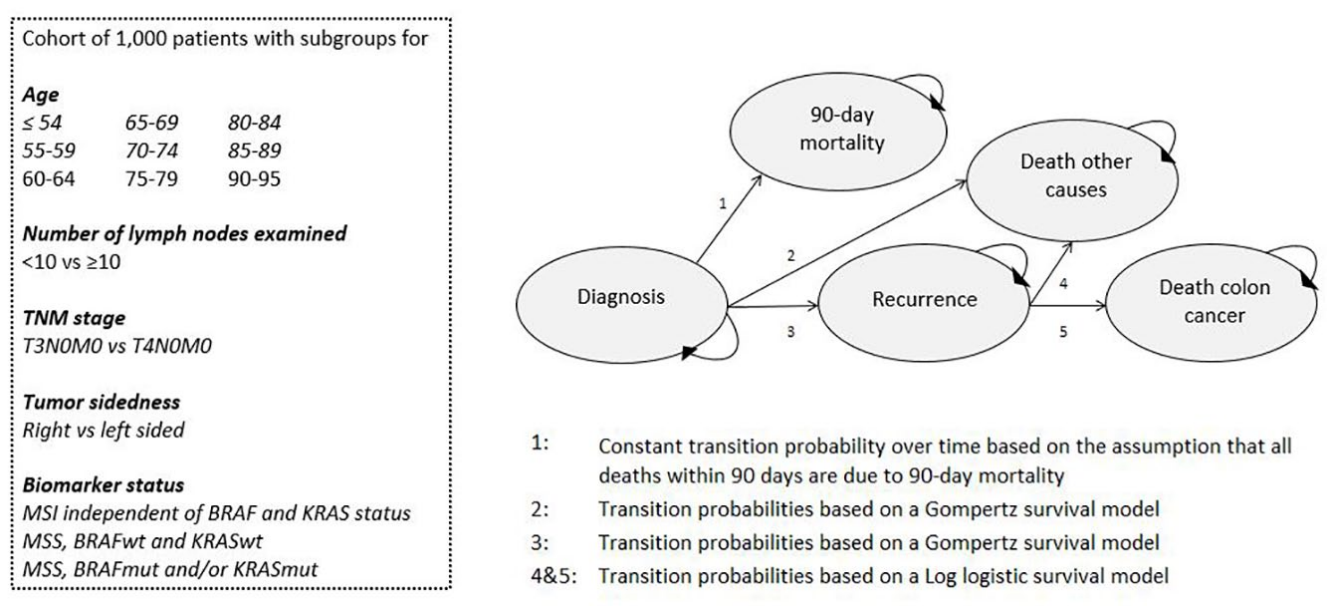
Flowchart of the Personalized Adjuvant TreaTment in EaRly stage coloN cancer (PATTERN) model. TNM, tumor–node–metastasis cancer staging.

The PATTERN model has been comprehensively described elsewhere.^16^ A flowchart of the model is shown in Figure 1 and model parameters are shown in Appendix Table A1. In short, the PATTERN model is a Markov cohort model with a lifelong time horizon and a 1-month cycle length. The model consists of five health states: diagnosis; 90-day mortality; death by other causes; recurrence; and death of colon cancer. Patient-level data from the NCR (*n* = 2271) and three external cohorts (*n* = 258) were used for model quantification.^17,18^ The PATTERN model was extensively validated both internally and externally. For the transition from diagnosis to 90-day mortality, we assumed that all observed deaths within 90 days after diagnosis were due to surgical complications. For all other transitions in the model, parametric survival models, including relevant covariates, were used for parametrization. Note that only patients without adjuvant chemotherapy treatment were included for fitting the survival models. In addition, we included an HR for treatment effect of 0.73 for fluoropyrimidine monotherapy combined with oxaliplatin, compared with no adjuvant treatment, based on pooled trial data (Appendix Table A1).^19^

In the model, 216 subgroups were distinguished based on number of lymph nodes evaluated (<10 and ⩾10), T stage (T3 and T4), tumor site (left and right), age (50–95 in nine 5-year categories), and biomarker status (microsatellite instability, MSS without a mutation in BRAF and KRAS and MSS combined with a mutation in BRAF and/or KRAS). The subgroups were weighted such that the distribution of clinical and pathological features reflects Dutch patients with colon cancer stage II. In line with the Dutch guideline recommendations, we only simulated high-risk patients (T4+MSS) in the current study.^4^

### Strategies

The base-case analysis was conducted for CAPOX and FOLFOX separately. In the reference strategy, high-risk patients received 6 months of oxaliplatin-based chemotherapy. Patients were classified as high risk according to the current Dutch guidelines (T4+MSS status).^4^ In the 6-month strategy, an HR for recurrence compared with no treatment of 0.73 was assumed for both CAPOX and FOLFOX, based on pooled trial data.^19^ Subsequently, a strategy was simulated in which high-risk patients receive 3 months of adjuvant chemotherapy. In the 3-month strategy, the above-mentioned HR for treatment effect in the 6-month strategy was multiplied with an HR for treatment effect for a 3-month during-treatment regimen compared with a 6-month during-treatment regimen, based on the pooled analysis for high-risk stage II colon cancer patients in the IDEA trials.^13,14^ These HRs for 3 *versus* 6 months of adjuvant chemotherapy were 1.0 (0.88; 1.17) for CAPOX (estimated in a population of 2019 high-risk stage II patients) and 1.4 (1.19; 1.70) for FOLFOX (estimated in a population of 1254 high-risk stage II patients). Note that the T4 stage, poor differentiation, invasion, inadequate nodal harvest, obstruction and perforation were specified as high-risk features in the IDEA trials.^10,12,13^

### Costs

In Table 1, an overview of resource use, costs, and utilities is presented. Costs were determined from a societal perspective and included costs for initial surgery, drugs, adverse events, absenteeism from work, patient travel to hospital, surveillance, and recurrence of disease.^4,6,7,20–25^ Costs of chemotherapy were calculated separately for CAPOX and FOLFOX. We assumed in the 6-month strategy that the FOLFOX regimen consisted of 12 cycles of 2 weeks, while the CAPOX regimen consisted of 8 cycles of 3 weeks.^4^ In the 3-month strategy, the number of cycles was halved for both CAPOX and FOLFOX. Per treatment schedule, we calculated the amount of medication needed for a patient with an average body surface of 1.7 m^2^. The adverse event rates were based on the TOSCA trial and were highest in the 6-month strategy.^26^ Per adverse event category, costs were based on follow-up care. For neutropenia, this was defined as a visit to the outpatient clinic for febrile neutropenia, as a hospital stay of 5 days, and for diarrhea, as oral rehydration medication.^20,22^ Surveillance was based on the Dutch guideline recommendations^4^ and consisted of consultations every half-year during the first 3 years after surgery, and yearly thereafter until 5 years after surgery. Each consultation is combined with an ultrasound scan of the liver, as well as a carcinoembryonic antigen (CEA) determination. In addition, patients undergo colonoscopy every 3 years, with the first colonoscopy 1 year after surgery.^4^

**Table 1.** Overview of resource use, unit costs and utilities. All costs were standardized to 2018 Euros, using the consumer price index.^27^

	6-month strategy	3-month strategy	Proportion 6 months/3 months	Reference
**Costs**				
Initial surgery	€12,987^a^	€12,987^a^		Nederlandse Zorgautoriteit^24^
Biomarker tissue test				Goldstein *et al*.^28^
MSI/IHC	€372	€372		
Treatment cost per full regimen				
CAPOX	€11,843	€5982		Adjuvante systemische therapie coloncarcinoom;^4^ Nederlandse zorgautoriteit;^20^ Hakkaart-van Roijen *et al*.^22^
% quitting before end regimen				7
FOLFOX	€19,563	€10,284		Adjuvante systemische therapie coloncarcinoom;^4^ Nederlandse zorgautoriteit;^20^ Hakkaart-van Roijen *et al*.^22^
% quitting before end regimen				7
Adverse event cost per case				
Grade 3/4 neutropenia	€95	€95	0.249/0.193^b^	Nederlandse zorgautoriteit;^20^ Ayvaci *et al*.;^21^ Hakkaart-van Roijen *et al*.;^22^ Lonardi *et al*.^26^
Febrile neutropenia	€3309	€3309	0.027/0.014^b^	Nederlandse zorgautoriteit;^20^ Ayvaci *et al*.;^21^ Hakkaart-van Roijen *et al*.;^22^ Lonardi *et al*.^26^
Grade 3/4 diarrhea	€50	€50	0.064/0.051^b^	Nederlandse zorgautoriteit;^20^ Ayvaci *et al*.;^21^ Hakkaart-van Roijen *et al*.;^22^ Lonardi *et al*.^26^
Absenteeism costs per cycle^c^				Hakkaart-van Roijen *et al*.^22^
<55	€5296	€5296		
55–65	€4911	€4911		
Travel costs per cycle	€8	€8		Hakkaart-van Roijen *et al*.^22^
Surveillance costs per patient^d^				
Colonoscopy	€850			Adjuvante systemische therapie coloncarcinoom;^4^ Vleugels *et al*.^23^
Colonoscopy with complications	€1430		0.028	Vleugels *et al*.^23^
Ultrasound scan	€83			Adjuvante systemische therapie coloncarcinoom;^4^ Hakkaart-van Roijen *et al*.^22^
CEA determination	€8			Adjuvante systemische therapie coloncarcinoom;^4^ Nederlandse Zorgautoriteit^24^
Relapse costs	€41,868			Tilson *et al*.^25^
**Utilities (95% CI)**				
Before surgery (month 1)	0.83 (0.82–0.84)	0.83 (0.82–0.84)		Iveson *et al*.;^10^ Robles-Zurita *et al*.^29^
After surgery/before chemotherapy (months 2–3)	0.81 (0.80–0.82)	0.81 (0.80–0.82)		Iveson *et al*.;^10^ Robles-Zurita *et al*.^29^
During chemotherapy (months 4–6)	0.81 (0.79–0.83)	0.86 (0.84–0.87)		Iveson *et al*.;^10^ Robles-Zurita *et al*.^29^
During/after chemotherapy (months 7–9)	0.83 (0.81–0.85)	0.87 (0.86–0.88)		Iveson *et al*.;^10^ Robles-Zurita *et al*.^29^
First year after chemotherapy (months 10–18)	0.85 (0.83–0.87)	0.88 (0.86–0.89)		Iveson *et al*.;^10^ Robles-Zurita *et al*.^29^
Years 2–5 after chemotherapy (months 19–60)	0.85 (0.82–0.87)	0.88 (0.87–0.90)		Iveson *et al*.;^10^ Robles-Zurita *et al*.^29^
More than 5 years after chemotherapy	0.90 (0.84–0.93)	0.90 (0.86–0.96)		Iveson *et al*.;^10^ Robles-Zurita *et al*.^29^
Recurrence months 1–60 after recurrence	0.45 (NA)	0.45 (NA)		Attard *et al*.;^30^ Ness *et al*.;^31^ van den Brink *et al*.^32^

a DBC tariffs from 24 hospitals in The Netherlands were averaged.

b Proportions apply across the entire 3-month or 6-month treatment regimen.

c To calculate the absenteeism costs we assumed that: (a) the male to female ratio was 0.47/0.53;^18^ (b) number of hours worked per week was 40 and 38 for men, and 28 and 25 for women in the age groups <55 and 55–65, respectively;^27^ and (c) patients do not work during chemotherapy. The absenteeism costs were calculated according to the friction-cost approach.

d Surveillance costs were calculated according to the Dutch guideline for colon cancer surveillance.

CAPOX, capecitabine plus oxaliplatin; CEA, cost-effectiveness acceptability; CI, confidence interval; FOLFOX, fluorouracil, leucovorin and oxaliplatin; IHC, immunohistochemistry; MSI, microsatellite instability; NA, not applicable.

### Health-related quality of life

To inform the model, we used utility estimates derived from the SCOT trial which reported the utilities for both a 3-month treatment regimen and 6-month treatment regimen.^10,29^ We calculated the average health utility in different time periods separately for the 3-month and 6-month strategy. We defined the following periods: before surgery; after surgery; before start of chemotherapy; during chemotherapy; first year after chemotherapy; 2–5 years after chemotherapy; and more than 5 years after chemotherapy (Table 1). As the health utility for patients who developed a recurrence was not reported in the SCOT trial, the utility for this health state was derived from other literature.^30–32^ Once a patient in the model developed a recurrence, the health utility was reduced to 0.45 for 60 months. At 60 months after recurrence, the patient was considered to be a cancer survivor and the value 0.90 was assigned, in line with the reported health utility in the SCOT trial for more than 5 years after chemotherapy. A full overview of health utilities is shown in Table 1.

### Outcome

Model outcomes consisted of the number of recurrences and deaths due to colon cancer per 1000 treated patients, life-years per patient (pp), quality-adjusted life-years (QALYs) pp, and total lifetime costs pp. Dutch discount rates of 4% and 1.5% were used for costs and health effects, respectively.^22^ The incremental net monetary benefit (NMB) of the 3-month strategy was calculated relative to the 6-month strategy as follows: [(incremental benefit × willingness to pay) − incremental cost], using a willingness-to-pay value of €50,000 per QALY. A positive incremental NMB indicates that the 3-month strategy is cost effective compared with the 6-month strategy. A negative incremental NMB indicates that the 3-month strategy is not cost effective compared with the 6-month strategy. Furthermore, the incremental cost-effectiveness ratio (ICER) was calculated as the ratio between the difference in costs and the difference in QALYs between the 3-month and 6-month strategies. A strategy is considered as cost effective when the ICER does not exceed the threshold value of €50,000 per QALY.^33^

### Sensitivity analysis

In the base-case analysis, we assumed in the 3-month strategy an HR for treatment effect for 3 months compared with 6 months’ chemotherapy of 1.0 (0.88; 1.17) for CAPOX, and 1.4 (1.19; 1.70) for FOLFOX. However, there is uncertainty around these treatment effects.^13,19^ To investigate the impact of this uncertainty on the base-case results, we performed a threshold analysis separately for CAPOX and FOLFOX. In this analysis, the HR for treatment effect for 3 months compared with 6 months chemotherapy for CAPOX was increased from 1.0 to 1.17 in accordance with the upper limit of the corresponding CI in the pooled IDEA analysis.^13^ For 3 months of FOLFOX, the HR decreased from 1.4 to 1.19, which is the lower limit of the CI for FOLFOX in the pooled IDEA analysis.^13^ Note that we only varied the treatment effect for the 3-month strategies in the direction in which changes could occur with regard to the conclusion of the cost effectiveness of the 3-month strategy compared with the 6-month strategy.

Furthermore, in a one-way sensitivity analysis, we investigated the impact of the uncertainty around the utility estimates derived from the SCOT trial. In this sensitivity analysis, we changed the health utilities according to the upper and lower limit of the CI reported in the SCOT trial (Table 1).^29^

Finally, a probabilistic sensitivity analysis was conducted to investigate the joint impact of parameter uncertainty. All parameters in the PATTERN model were varied simultaneously according to the most appropriate distribution. The Monte Carlo simulation, which was conducted separately for CAPOX and FOLFOX, consisted of 1000 iterations for both the 3-month strategy and the 6-month strategy. To graphically illustrate the uncertainty surrounding the base-case model predictions, incremental costs and effects were plotted on a cost-effectiveness plane. In addition, cost-effectiveness acceptability curves (CEACs) were constructed for CAPOX and FOLFOX separately.

## Results

### Effectiveness

In the 6-month strategy, the model predicted 369 recurrences and 316 colon cancer deaths for both CAPOX and FOLFOX in the lifetime of 1000 high-risk stage II colon cancer patients. For CAPOX, these figures for the 3-month strategy were equal to the 6-month strategy. For FOLFOX, the model predicted 457 recurrences and 393 colon cancer deaths in the 3-month strategy in a cohort of 1000 patients, which corresponds to an increase of 19.3% and 19.6% compared with the 6-month strategy, respectively (Table 2). For both CAPOX and FOLFOX, the predicted QALYs in the 6-month strategy were 6.71 pp. For CAPOX, the predicted QALYs increased to 6.80 pp in the 3-month strategy, while for FOLFOX the QALYs decreased to 6.19 pp.

**Table 2.** Base-case results of the cost-effectiveness analysis.

	Colon cancer burden^a^		LYs per treated patient (years)		QALYs per treated patient (years)		Costs per treated patient (€)		Incremental NMB^b^	ICER in €/QALY (quadrant)
	Recurrences	Deaths	Undiscounted	Discounted	Undiscounted	Discounted	Undiscounted	Discounted		
CAPOX										
6-month strategy	369	316	9.09	8.05	7.60	6.71	43,328	41,257	Reference	Reference
3-month strategy	369	316	9.09	8.05	7.60	6.80	39,640	37,645	8454	−37,308 (SE)^c^
FOLFOX										
6-month strategy	369	316	9.09	8.05	7.60	6.71	49,301	47,135	Reference	Reference
3-month strategy	457	393	8.36	7.43	6.99	6.19	46,632	44,389	−23,189	5293 (SW)

a Total during the lifetime of a cohort of 1000 treated patients.

b At a willingness to pay of €50,000/QALY

c ICER indicates that the 3-month strategy is more effective and less costly compared with 6 months. In other words, you save €37,308 to gain 1 QALY.

CAPOX, capecitabine plus oxaliplatin; FOLFOX, fluorouracil, leucovorin and oxaliplatin; ICER, incremental cost-effectiveness ratio; LYs, life-years; NMB, net monetary benefit; QALYs, quality-adjusted life-years; SE, the strategy is located in the southeast quadrant of the cost-effectiveness plane, indicating that a strategy is less costly and more effective compared with the reference strategy; SW, the strategy is located in the southwest quadrant of the cost-effectiveness plane, indicating that a strategy is less costly and less effective compared with the reference strategy.

### Cost effectiveness

For CAPOX, the predicted costs pp were €41,257 for the 6-month strategy and €37,645 for the 3-month strategy. This cost difference of €3612 was due to saving resources related to the adjuvant chemotherapy. The incremental NMB for the 3-month strategy relative to the 6-month strategy was €8454, considering a willingness to pay of €50,000/QALY. The 3-month strategy was more effective and less costly compared with the 6-month strategy, leading to a negative ICER of −€37,308/QALY. This indicates that the 3-month strategy is cost saving compared with the 6-month strategy.

For FOLFOX, the predicted costs were €47,135 for the 6-month strategy and €44,389 for the 3-month strategy. An incremental NMB of −€23,189 was found for the 3-month strategy compared with the 6-month strategy, indicating that the 3-month strategy is not cost effective compared with the 6-month strategy.

### Sensitivity analysis

**Figure 2.**
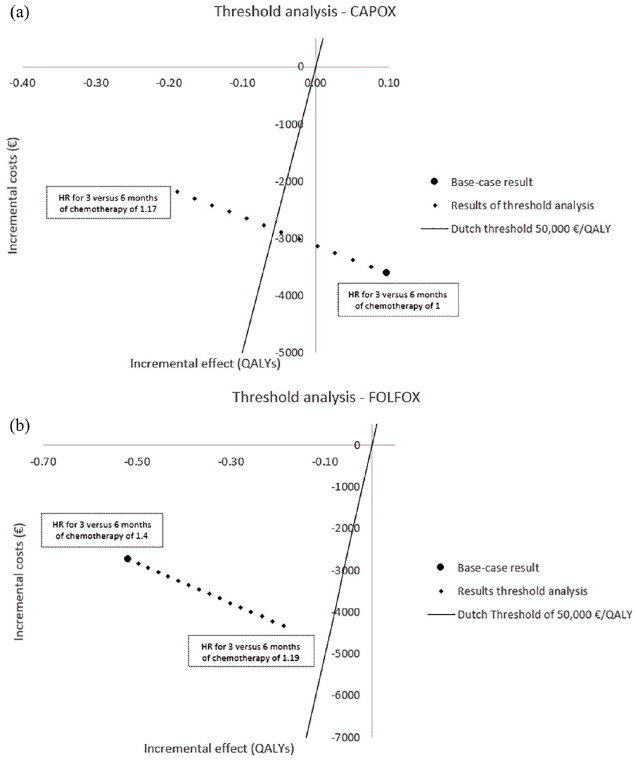
Results of the threshold analysis for CAPOX and FOLFOX. Results of the threshold analysis for CAPOX (a) in which the HR for treatment effect for 3 *versus* 6 months of adjuvant chemotherapy was increased from 1.0 to 1.17, and FOLFOX (b) in which the HR for treatment effect for 3 *versus* 6 months of adjuvant chemotherapy decreased from 1.4 to 1.19. The figure is shown in comparison with the 6-month strategy, so the origin of the curve represents the situation for 6 months. The solid line through the origin corresponds to an ICER of €50,000/QALY. Therefore, every result below that line is considered cost effective and everything above the line is considered not cost effective. CAPOX, capecitabine plus oxaliplatin; FOLFOX, fluorouracil, leucovorin and oxaliplatin; HR, hazard ratio; ICER, incremental cost-effectiveness ratio; QALY, quality-adjusted life-year.

Results of the threshold analysis, in which treatment effect for the 3-month strategy was varied separately for CAPOX and FOLFOX, are shown in Figure 2. Note that the HR for recurrence for the 6-month strategy compared with no treatment remained the same as in the base-case analysis, namely 0.73. For CAPOX, the 3-month strategy is no longer delivering more QALYs than the 6-month strategy when the HR for a 3-month strategy compared with a 6-month strategy is higher than 1.06. Furthermore, the 3-month strategy is no longer cost effective compared with the 6-month strategy when the HR for a 3-month strategy compared with a 6-month strategy is higher than 1.09 [Figure 2(a)]. For FOLFOX, the 3-month strategy was not more effective nor cost effective compared with the 6-month strategy when we assumed an HR for a 3-month strategy compared with a 6-month strategy in a range of 1.19–1.4 [Figure 2(b)].

Results of the one-way sensitivity analysis, in which we changed the utility estimates according to the upper and lower limit of the confidence intervals derived from the SCOT trial, are reported in Table 3. The results of this sensitivity analysis were comparable with the results of the base-case analysis.

**Table 3.** Results of the one-way sensitivity analysis, in which we varied the utility estimates according to the upper and lower limits of the confidence intervals reported in the SCOT trial.^29^

	Colon cancer burden^a^		LYs per treated patient	QALYs per treated patient	Costs per treated patient (€)	Incremental NMB^b^	ICER in €/QALY (quadrant)
	Recurrences	Deaths	Discounted	Discounted	Discounted		
Utility estimates according to the lower limit of the SCOT trial							
CAPOX							
6-month strategy	369	316	8.05	6.46	41,257	Reference	Reference
3-month strategy	369	316	8.05	6.68	37,645	14,746	minus 16,224 (SE)
FOLFOX							
6-month strategy	369	316	8.05	6.46	47,135	Reference	Reference
3-month strategy	457	393	7.43	6.08	44,389	−16,226	7236 (SW)
Utility estimates according to the upper limit of the SCOT trial							
CAPOX							
6-month strategy	369	316	8.05	6.98	41,257	Reference	Reference
3-month strategy	369	316	8.05	7.19	37,645	14,240	−16,998 (SE)
FOLFOX							
6-month strategy	369	316	8.05	6.98	47,135	Reference	Reference
3-month strategy	457	393	7.43	6.53	44,389	−19,746	6103 (SW)

a Total during the lifetime of a cohort of 1000 treated patients.

b At a willingness to pay of €50,000/QALY.

CAPOX, capecitabine plus oxaliplatin; FOLFOX, fluorouracil, leucovorin and oxaliplatin; ICER, incremental cost-effectiveness ratio; LYs, life-years; NMB, net monetary benefit; QALY, quality-adjusted life-year; SE, the strategy is located in the southeast quadrant of the cost-effectiveness plane, indicating that a strategy is less costly and more effective compared with the reference strategy; SW, the strategy is located in the southwest quadrant of the cost-effectiveness plane, indicating that a strategy is less costly and less effective compared with the reference strategy.

### Probabilistic sensitivity analysis

**Figure 3.**
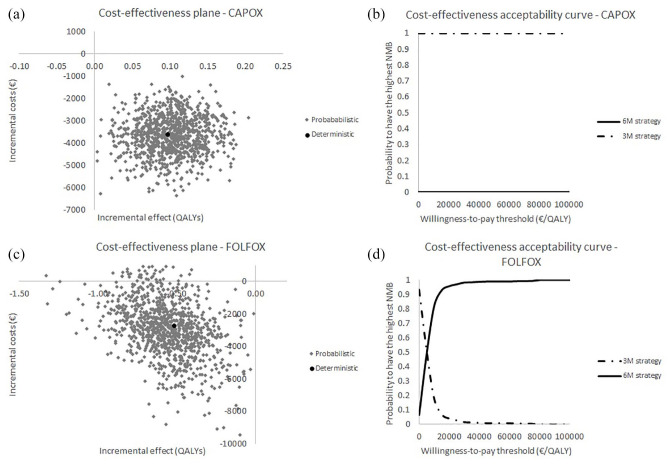
Results of the probabilistic sensitivity analysis. Results of the probabilistic sensitivity analysis presented in a cost-effectiveness plane, and cost-effectiveness acceptability curve, separately for CAPOX (a, b) and FOLFOX (c, d). CAPOX, capecitabine plus oxaliplatin; FOLFOX, fluorouracil, leucovorin and oxaliplatin; QALY, quality-adjusted life-years.

Figure 3 presents the results of the probabilistic sensitivity analysis. For CAPOX, the 3-month strategy is more effective and less costly compared with the 6-month strategy [Figure 3(a)]. The CEAC showed that the probability of having the highest NMB was 1.0 for the 3-month strategy and 0 for the 6-month strategy in a range of willingness-to-pay thresholds of €0–100,000 [Figure 3(b)]. For FOLFOX, the 3-month strategy is less effective and less costly compared with the 6-month strategy [Figure 3(c)]. The CEAC showed that the probability of having the highest NMB was highest for the 3-month strategy up to a willingness-to-pay threshold of €10,000/QALY. For a willingness to pay above €10,000/QALY, the probability to have the highest NMB was higher for the 6-month strategy than for the 3-month strategy. At a willingness to pay of €50,000 €/QALY, the probability that the 6-month strategy results in the highest NMB is 0.99.

## Discussion

This study evaluated the cost effectiveness of a 3-month adjuvant chemotherapy regimen compared with a 6-month chemotherapy regimen consisting of either FOLFOX or CAPOX in high-risk stage II colon cancer patients using the PATTERN model.^16^ For both CAPOX and FOLFOX, the 3-month strategy was less costly compared with the 6-month strategy (cost difference €3612 and €2745 per person, respectively). The lower costs were mainly caused by cost savings related to adjuvant chemotherapy. For CAPOX, the QALY lived were highest in the 3-month strategy (0.1 QALY difference pp), resulting in an incremental NMB of €8454 at a willingness-to-pay threshold of €50,000/QALY. For FOLFOX, QALYs lived were lower in the 3-month strategy compared with the 6-month strategy (0.5 QALY difference pp), resulting in a negative incremental NMB of €23,189 at a willingness-to-pay threshold of €50,000/QALY. The robustness of the base-case results was underpinned by a probabilistic sensitivity analysis.

The divergent results for CAPOX and FOLFOX were mainly due to the difference in the assumed HR for 3 *versus* 6 months of adjuvant chemotherapy, which was based on a pooled analysis of the IDEA trials. For CAPOX, we assumed an HR of 1.0, which amounts to an equal treatment effect compared with no adjuvant treatment as in the 6-month strategy. For FOLFOX, we assumed an HR of 1.4, which is equal to no effect of adjuvant treatment at all. To investigate the influence of uncertainty around the pooled HRs from the IDEA collaboration on our base-case results, a threshold analysis was conducted separately for CAPOX and FOLFOX. For 3 months’ CAPOX, the results showed a decrease in QALYs compared with 6 months’ CAPOX when the HR for 3 *versus* 6 months of adjuvant chemotherapy changed from 1.00 to 1.06 or higher. Given the relatively small change in HR that would lead to a QALY loss instead of gain, it would be appropriate to reappraise the cost effectiveness of a 3-month CAPOX regime when additional data on the comparison of a 3-month regimen with a 6-month regime becomes available. For FOLFOX, the 3-month strategy resulted in lower QALYs than the 6-month strategy, for the entire range of HRs that was assumed in the threshold analysis. These findings indicate that the 3-month regimen for FOLFOX should not be recommended in clinical practice, given current data.

The uncertainty in the trial data that we used as input for our analyses should also be considered in daily clinical practice, as discussed in the work from Moretto *et al*.,^14^ especially because the non-inferiority for a 3-month strategy compared with a 6-month strategy, for both FOLFOX and CAPOX, was not proven in a per-protocol analysis among stage III colon cancer patients.^34^ A per-protocol analysis is recommended to perform in non-inferiority trials because the usual intention-to-treat analysis may be biased by the inclusion of patients who did not follow the protocol sufficiently.^35^ Unfortunately, no per-protocol sub-analysis was conducted for stage II colon cancer patients. Nevertheless, given the conflicting results between the intention-to-treat and per-protocol analysis in stage III colon cancer patients, we should interpret the results of the current study carefully. As Moretti *et al.* propose, after the first 3 months of CAPOX, it would be appropriate to discuss treatment continuation per individual patient such that the patient’s preference, tolerance, and degree of neurotoxicity are taken into account.^14^

To our knowledge, this is the first model-based study evaluating the cost effectiveness of a 3-month during-treatment regimen compared with a 6-month during-treatment regimen in high-risk stage II colon cancer patients only. An evaluation of the cost effectiveness of a 3-month strategy compared with a 6-month strategy in high-risk stage II and III colorectal cancer patients was conducted earlier for the SCOT trial by Robles-Zurita *et al*.^29^ and Iveson *et al*.^10,36^ These studies concluded that a 3-month regimen with either FOLFOX or CAPOX was cost effective compared with a 6-month regimen, and that a 3-month regimen should be considered as a new standard of care for both FOLFOX and CAPOX.^29^ This conclusion is in line with the findings of our study for CAPOX, but in contrast to our findings for FOLFOX. These contrasting results for FOLFOX can be explained by the HR of 1.16 for the 3-month FOLFOX regimen compared with the 6-month FOLFOX regimen reported in the SCOT trial. As mentioned above, we assumed an HR for 3 months compared with 6 months’ adjuvant chemotherapy of 1.4 in the current study, based on pooled data of the IDEA collaboration.^13^ Nevertheless, in the FOLFOX subgroup analysis of the SCOT trial, authors found 0.1 lower QALYs pp in the 3-month strategy compared with the 6-month strategy, which is in line with the findings of our study. However, in the SCOT trial, the cost savings for the 3-month FOLFOX regimen compared with the 6-month FOLFOX regimen were such that a higher NMB was found for the 3-month regimen compared with the 6-month regimen. A second explanation for the different directions of the results for FOLFOX could be a difference in study population. To illustrate, only 18.1% of the patients included in the SCOT trial were classified as stage II. Further comparison of the study populations was hampered, because separate baseline characteristics for stage II patients were not presented for the SCOT trial.^10,29^

In the current study, we included an HR for treatment effect for the 6-month strategy of 0.73 for FOLFOX compared with no adjuvant chemotherapy, based on pooled trial data.^19^ Due to a lack of available trial data, no separate HR was reported in this study for CAPOX compared with no adjuvant treatment. Therefore, we assumed in the current study that the HR for treatment effect is similar for CAPOX and FOLFOX for the 6-month regimen, which is justified, based on literature in stage III colon cancer.^37–39^ To illustrate, the X-ACT trial showed that capecitabine is non-inferior to fluorouracil. Subsequently, the NO169968 trial showed that the benefit of adding CAPOX is similar to the addition of FOLFOX.

The results of our economic evaluation support the adjustment in the Dutch guidelines in which the duration for a CAPOX regimen is shortened from 6 months to 3 months for high-risk stage II colon cancer patients (MSS+T4). However, guideline adherence in clinical practice may be suboptimal, as has been shown in The Netherlands in both stage II and III colon cancer.^40^ Possible explanations for this suboptimal adherence are differences in expert opinions and unawareness of the guidelines. Based on these findings, extra support for guideline implementation and monitoring in clinical practice should be considered. Furthermore, in The Netherlands, approximately 95% of stage II colon cancer patients who receive adjuvant treatment receive CAPOX, leading to a relatively high benefit for society if the treatment duration of CAPOX is shortened. However, internationally, FOLFOX is prescribed in the majority of cases. To illustrate, in the IDEA studies, FOLFOX was prescribed to 90% of the patients in France, 65% of patients in Italy and 42% to the patients in Greece.^34^ Our results do not support a duration of 3 months for FOLFOX, as this leads to more recurrences and fewer QALYs.

In line with the IDEA collaboration, our study focused on comparing a 6-month treatment duration with a 3-month treatment duration rather than comparing FOLFOX and CAPOX.^10,12,13^ However, the results reported in the current study strongly suggest that 3 months of CAPOX is cost effective compared with both 3 and 6 months of FOLFOX, given the higher QALYs and lower costs. The associated incremental NMBs are −37,521 and −14,332 for 3 months CAPOX compared with 3 and 6 months FOLFOX, respectively, using a threshold value of €50,000/QALY.

In conclusion, this is the first model-based study that evaluated the cost effectiveness of a 3-month adjuvant chemotherapy regimen compared with a 6-month chemotherapy regimen, separately for CAPOX and FOLFOX, in high-risk stage II colon cancer. Our findings indicate that a 3-month regimen is the most favorable treatment duration for CAPOX, given the higher QALYs and lower costs compared with 6 months. Nevertheless, given the uncertainty in the data we used as input for our analyses, the decision to continue the treatment after the first 3 months should be considered per individual based on the patient’s attitude, tolerance, and degree of neurotoxicity. For FOLFOX, our findings showed that 6 months of adjuvant chemotherapy is the optimal duration.

## Supplemental Material

Appendix – Supplemental material for Model-based evaluation of the cost effectiveness of 3 versus 6 months’ adjuvant chemotherapy in high-risk stage II colon cancer patients Supplemental material, Appendix for Model-based evaluation of the cost effectiveness of 3 versus 6 months’ adjuvant chemotherapy in high-risk stage II colon cancer patients by Gabrielle Jongeneel, Marjolein J. E. Greuter, Felice N. van Erning, Miriam Koopman, Geraldine R. Vink, Cornelis J. A. Punt and Veerle M. H. Coupé in Therapeutic Advances in Gastroenterology
