# Supplementary material for: Model-based evaluation of the cost effectiveness of 3 versus 6 months’ adjuvant chemotherapy in high-risk stage II colon cancer patients
Source: Ther Adv Gastroenterol. 2020 Sep 16;13:1756284820954114. doi: 10.1177/1756284820954114 (PMC7502861; doi:10.1177/1756284820954114)
Supplement: Appendix – Supplemental material for Model-based evaluation of the cost effectiveness of 3 versus 6 months’ adjuvant chemotherapy in high-risk stage II colon cancer patients [file Appendix.pdf]

Appendix Table 1. Parameter estimates specifying transitions in the PATTERN model.

	Transition Diagnosis to 90DM (DIAG-90DM)	Transition Diagnosis to DOC (DIAG-DOC)		Transition Diagnosis to recurrence (DIAG-REC)		Transition Recurrence to Death (REC-DOC + REC-DCC)[d]		Transition Recurrence to DOC (REC-DOC)
Parametric distribution	NA	Gompertz		Gompertz		Log Logistic		NA
	Probability	Coefficient (95%CI)	P-value	Coefficient (95%CI)	P-value	Coefficient (95%CI)	P-value	Probability
Shape (intercept)	NA	0.010 (0.009;0.012)	<0.01	-0.016 (-0.021;-0.010)	<0.01	1.17 (1.06;1.29)	<0.01	NA
Rate / Scale	NA	0.000 (0.000;0.001)	0.02	0.004 (0.003;0.005)	<0.01	1,390 (397;4,850)	0.48	NA
Age								
≤ 54	0.028 (0.004;0.052)	Reference				-3.439 (-4.363;-2.515)		0.000
55-59	0.028 (0.004;0.052)	0.632 (-0,045;1,308)				-3.766 (-4.778;-2.754)		0.001
60-64	0.032 (0.011;0.053)	0.591 (-0.037;1,219)				-4.094 (-5.194;-2.994)		0.001
65-69	0.032 (0.011;0.053)	1.039 (0.453;1.624)				-4.421 (-5.609;-3.233)		0.001
70-74	0.064 (0.040;0.087)	1.701 (1.150;2.252)	<0.01	NA	NA	-4.749 (-6.025;-3.473)	<0.01	0.002
75-79	0.073 (0.050;0.097)	2.159 (1.617;2.702)				-5.076 (-6.440;-3.712)		0.003
80-84	0.114 (0.081;0.147)	2.823 (2.281;3.365)				-5.404 (-6.856;-3.952)		0.004
85-89	0.156 (0.099;0.213)	3.166 (2.604;3.729)				-5.731 (-7.271;-4.191)		0.005
90-95	0.333 (0.178;0.488)	3.315 (2623;4.007)				-6.059 (-7.687;-4.431)		0.006
Lymph nodes evaluated (≥10 vs <10)	NA	NA	NA	-0.519 (-0.762;-0.276)	<0.01	NA	NA	NA
pT stage (pT4 vs pT3)	NA	NA	NA	1.081 (0.779;1.383)	<0.01	NA	NA	NA
Tumor side (Left vs right)	NA	NA	NA	0.505 (0.272;0.737)	<0.01	NA	NA	NA
Biomarker subgroup								
MSI				-1.398[a] (-2.571;-0.224)	0.14			
MSSdwt	NA	NA	NA	-0.128[a] (-1.033;0.776)	1.00	NA	NA	NA
MSSmut				0.424[a] (-0.575;1.423)	0.95			
Treatment effect	NA	NA	NA	-0.250[a,b] (-0.383;-0.118)	<0.01	NA	NA	NA
	NA	NA	NA	-0.063[a,c] (-0.276;0.149)	0.13	NA	NA	NA

Abbreviations: 90DM = 90-day mortality; DIAG = diagnosis; DOC = death other causes; REC = recurrence; DCC = death of colon cancer; NA = not applicable. [a] Parameters used in the model are the log transformations of the estimated hazard ratios of 0.247, 0.880, 1.528, 0.779, 0.939 respectively for MSI, MSSdwt, MSSmut and treatment effect. [b] Treatment effect for

fluoropyrimidine monotherapy compared to no adjuvant chemotherapy. [c] Treatment effect for FOLFOX compared to Fluoropyrimidine monotherapy. [d] Transition REC-DOC and REC-DCC were estimated in the same parametric survival model. Transition REC-DCC is calculated as the difference of transition REC-DOC + REC-DCC and transition REC-DOC.
